# Enhanced Periosteal and Endocortical Responses to Axial Tibial Compression Loading in Conditional Connexin43 Deficient Mice

**DOI:** 10.1371/journal.pone.0044222

**Published:** 2012-09-10

**Authors:** Susan K. Grimston, Marcus P. Watkins, Michael D. Brodt, Matthew J. Silva, Roberto Civitelli

**Affiliations:** 1 Division of Bone and Mineral Diseases, Department of Internal Medicine, Washington University in St. Louis, St. Louis, Missouri, United States of America; 2 Department of Orthopedic Surgery, Washington University in St. Louis, St. Louis, Missouri, United States of America; 3 Musculoskeletal Research Center, Washington University in St. Louis, St. Louis, Missouri, United States of America; University of Notre Dame, United States of America

## Abstract

The gap junction protein, connexin43 (Cx43) is involved in mechanotransduction in bone. Recent studies using in vivo models of conditional Cx43 gene (*Gja1*) deletion in the osteogenic linage have generated inconsistent results, with *Gja1* ablation resulting in either attenuated or enhanced response to mechanical load, depending upon the skeletal site examined or the type of load applied. To gain further insights on Cx43 and mechanotransduction, we examined bone formation response at both endocortical and periosteal surfaces in 2-month-old mice with conditional *Gja1* ablation driven by the *Dermo1* promoter (cKO). Relative to wild type (WT) littermates, it requires a larger amount of compressive force to generate the same periosteal strain in cKO mice. Importantly, cKO mice activate periosteal bone formation at a lower strain level than do WT mice, suggesting an increased sensitivity to mechanical load in Cx43 deficiency. Consistently, trabecular bone mass also increases in mutant mice upon load, while it decreases in WT. On the other hand, bone formation actually decreases on the endocortical surface in WT mice upon application of axial mechanical load, and this response is also accentuated in cKO mice. These changes are associated with increase of *Cox-2* in both genotypes and further decrease of *Sost* mRNA in cKO relative to WT bones. Thus, the response of bone forming cells to mechanical load differs between trabecular and cortical components, and remarkably between endocortical and periosteal envelopes. Cx43 deficiency enhances both the periosteal and endocortical response to mechanical load applied as axial compression in growing mice.

## Introduction

Connexin43 (Cx43) is the predominant gap junction protein present in bone cells, particularly in cells of the osteoblast lineage [1]–[3]. The importance of Cx43 for bone homeostasis is demonstrated by the skeletal abnormalities present in mice with ablation of the Cx43 gene (*Gja1*) [4], and further underscored by the skeletal malformations described in patients with the autosomal dominant disorder oculodentodigital dysplasia, a disease linked to *Gja1* mutations [5]–[7].

We have previously reported that mice with conditional deletion of *Gja1* in cells of the osteogenic lineage have enlarged marrow cavities, thin cortices and hypomineralized long bones [8]–[11]. We also find that activation of endocortical bone formation by application of mechanical load in vivo by a 3-point bending regimen is attenuated in Cx43 deficient mice driven by a 2.3 kb fragment of the *Col1a1* promoter [9]. In that study, periosteal response to the mechanical load was not quantifiable because of exuberant production of woven bone on the periosteal surface of both Cx43 deficient and wild type mice, likely representing a reaction to compressive pressure generated by the device used to produce the load [9]. More recently, others have used cantilever bending to apply load in vivo, and found actually enhanced periosteal bone formation response in *Gja1* deleted mice driven by the *Bglap* promoter [12]. Endocortical response was not reported in that study. While the different methods used to apply skeletal load, the different anatomical locations studied, and perhaps even the slightly different genetic models of *Gja1* ablation may contribute to explain the apparently discrepant result, it is also quite conceivable that endocortical and periosteal bone cells respond differently to mechanical load, a discrepancy that may be affected or even enhanced by Cx43 deficiency.

In this study, we addressed this issue by applying a more physiologic loading regime, axial tibial compression, and evaluated the bone formation response at both periosteal and endocortical surfaces. We also tested whether the altered response of Cx43 deficient bones is related to abnormal sensitivity to load, and examined the gene expression profile in bones before and after loading to garner some insights on the potential mechanisms of Cx43 modulation of mechanical skeletal responses. For these studies, we used mice in which the *Dermo1* promoter drives *Gja1* deletion in the chondro-osteogenic lineage, and whose phenotype of larger but thinner cortical bone is accentuated relative to other genetic models of more restricted *Gja1* deletion. We reasoned that any roles that Cx43 may have in mediating the osteo-anabolic response to mechanical load should be more evident in these mice with a broader *Gja1* ablation.

Our results indicate that Cx43 deficiency alters the endocortical and periosteal responses to mechanical load, resulting in enhanced activation of periosteal bone formation and further decrease in endocortical bone formation induced by axial tibial compression. We also find Cx43-dependent altered expression of selected genes involved in mechanotransduction in bone.

## Methods

### Ethics Statement

This study was carried out in strict accordance with the recommendations in the Guide for the Care and Use of Laboratory Animals of the National Institutes of Health. All procedures were approved by the Animal Studies Committee of Washington University in St. Louis (protocol number 20090128).

### Transgenic Mice

Mice were generated as described previously [10]. Briefly, mice harboring a mutant “floxed” *Gja1* allele were mated to mice expressing *Cre* under the control of the *Dermo1/Twist2* promoter resulting in *Cre*-mediated gene replacement of the entire *Gja1* reading frame with the *LacZ* reporter cassette. One Gja1 null allele was also introduced to generate *DM1-Cre;Gja1^flox/−^* conditional knockout (cKO) mice. *Gja1^flox/+^* were used as wild type equivalent (WT) mice. The *DM1-Cre* transgene effectively deletes *Gja1* in osteoblasts on the endocortical surface, osteocytes [10], as well as periosteal cells (Figure S1). Genotyping was performed by PCR on genomic DNA extracted from mouse tails using the HotSHOT method [13]. Primers and conditions have been described previously [10]. All mouse lines were developed in a mixed C57BL/6-C129/J background and cKO and WT littermates were used. Mice were fed regular chow and water ad libitum and housed communally in a room maintained at a constant temperature (25°C) on a 12 hour light/dark schedule. Mice were weighed daily during the loading phase of the study as an index of overall body health.

### Strain Gauge Analysis

Strain-force relationship studies were performed on 2-month-old male cKO (n = 8) and WT (n = 6) mice using tibial compression loading as described previously [14]. Briefly, after euthanasia 1 mm strain gauges (Tokyo Sokki Kenkyujo Co., Japan) were placed along the longitudinal axis of the antero-medial surface of the right tibia, at a point 5 mm proximal to the distal tibiofibular junction (TFJ). Previous analysis indicated that the site of peak axial tensile strain is at this location [15]. Because of the curvature of the tibia axial compression generates combined compression-bending at the aforementioned point. Mice were positioned in a servohydraulic materials testing machine (Instron; Dynamite) such that the loading piston applied a compressive load through contacts at the knee and ankle (foot up, knee down) [14]. After application of a 0.5 N compressive pre-load, a trapezoidal waveform was applied to generate peak forces of 4 N up to a maximum of 14 N, at 2 N intervals. A 10 sec rest interval was allowed between each of 8 loading cycles for each force tested. Strains were recorded through a signal conditioning amplifier system (SCX1-1001; National Instruments), with 160 secs recovery interval between each set of eight cycles. Peak strains were determined for cycles 4–8, and the average computed. The region of the bone at the gauge site, with the strain gauge attached was scanned using µCT (µCT- 40; Scanco, Basserdorf, Switzerland) to assess the location of the strain gauge and the accuracy of its placement relative to the TFJ. From cross sectional images, the bone centroid, area, and moment of inertia about the neutral axis were determined as well as the distance from the centroid to the gauge location (y_gauge_) and the predicted site of peak periosteal strain (y). Based on beam theory [16], we then extrapolated the strain-gauge data to estimate strain values at the site of maximal tensile periosteal strain (ε) using the relation ε = ε_gauge_(y/y_gauge_).

### In Vivo Axial Compression Loading

Mice (2 month old) were loaded in axial tibial compression using the Instron Dynamite, 5 consecutive days, 60 cycles per day, with a 10 sec rest interval inserted between each loading cycle, as described previously [14]. After a 0.5 N pre-load was applied, a trapezoidal waveform was applied at 40 N/sec. Peak forces were chosen based on the strain analysis (see Results). The controlateral tibia was used as an internal, non-loaded control in all cases. Mice were loaded under isofluorane and were kept in isolation until full recovery from anesthesia. Mice were given a subcutaneous injection of Buprenex (0.1 ml/25 mg) following each loading session; they were allowed normal ambulatory cage activity between loading sessions and from the end of the loading period until sacrifice. Mice were injected with calcein on day 4 (4 µl/g body weight) and with Alizarin Red (6 µl/g body weight) on day 9, and were sacrificed on day 11. For gene expression analysis, 2-month-old WT and cKO mice were all loaded at a force of 7 N, using 120 cycles with a 10 second rest interval between cycles. Mice were sacrificed 2 hours after the loading session for RNA extraction (see below).

### In Vivo µCT

In vivo µCT scans were performed as described previously [11], [14], using a VivaCT 40 scanner (Scanco Medical). Briefly, mice were anesthetized with isoflurane and positioned in a specifically designed fixture providing for reliable placement of the leg. Scan settings were 70 KVp, 109 µA and 21.5 µm voxel resolution. Cortical bone parameters were assessed at a point 5 mm proximal to the TFJ (25 slices); trabecular bone was analyzed as the area immediately distal to the growth plate (100 slices) in the metaphysis. Contours were drawn manually of the area encompassing the trabecular bone and excluding the cortical shell.

### Histomorphometry

Bone formation indices were assessed using standard histomorphometric techniques [17]. Tibiae were fixed in 10% buffered formalin for up to 24 hours, dehydrated and embedded in methyl methacrylate as described previously [9]. Triplicate 100 µm thick transverse sections were cut (Leica 1600SP, Wetzlar, Germany) from each tibia 5 mm proximal to the TFJ. Sections were mounted on glass and polished to a thickness of approximately 30 µm. Two color fluorescent images were taken (calcein – green, and alizarin – red) using a confocal microscope (LSM 510, Axiovert 200 M, Plan-Neofluar Carl Zeiss, Jena, Germany). Dynamic histomorphometry analysis was performed using commercially available software (OSTEO II, Bioquant, Nashville, TN, USA). Single and double labeling surface (sLS/BS, dLS/BS), mineral apposition rate (MAR) and bone formation rate (BFR/BS) were determined as defined previously [9], [17]. Endocortical and periosteal surfaces were analyzed separately. Single labeled surface was defined as any surface with green, red, or yellow (no separation between green and red). Double labeled surface was any surface with green and red separated by a measureable distance. MS/BS was calculated as 0.5× sLS/BS + dLS/BS for all samples. Based on a previously published recommendation [18] samples with no dLS/BS were coded as “no data” for MAR and BFR/BS. Some sections were decalcified and processed for β-galactosidase staining as detailed [8], with the addition of 100 mM galactose to the staining solution to quench non-specific staining.

### Gene Expression Analyses

Tibiae were cleaned of all connective tissue and after dissection of the distal epiphysis, the bone marrow was removed by centrifugation (10,000 rpm for 15 sec). Tibiae were then flash frozen in liquid nitrogen for storage at −80°C. The bones were pulverized using a Braun Mikrodismembranator (Sartorius BBI Systems Inc., Bethlehem, PA) and total RNA was extracted using Trizol reagent (Invitrogen, Carlsbad, CA) and purified using Phase-Lock gel tubes (5 Prime, Hamburg, Germany) and the RNeasy Mini Kit (Qiagen) according to the manufacturer's directions. DNA was removed using the RNase-free DNase set (Qiagen, Valencia, CA). Messenger RNA was measured and 1 µg total RNA was reverse transcribed using a superscript VILO cDNA synthesis kit (Invitrogen, Carlsbad, CA). The SYBR green (Applied Biosystems, Foster City, CA) realtime PCR method was used in an Applied Biosystem 7500 Fast detector system, as described previously [19], [20]. PCR products were normalized to cyclophilin (*Ppib)*. Primer sets are detailed in Table S1.

### Statistical Analysis

Differences between groups were assessed by paired t-tests (two-tailed). Effects of load and genotype were assessed using two-way ANOVA, with post-hoc comparisons using the Bonferroni multiple t-test (SigmaPlot Version 11.0; Systat Software GmbH, Germany). Where the data indicated, one-way ANOVA was applied to test for differences among groups. Significant differences between slopes of Force-Strain regression lines were determined using the Test for Parallelism. Differences were defined as statistically significant at p<0.05. Values are expressed as the mean ± standard error of the mean unless otherwise specified.

## Results

**Figure 1:**
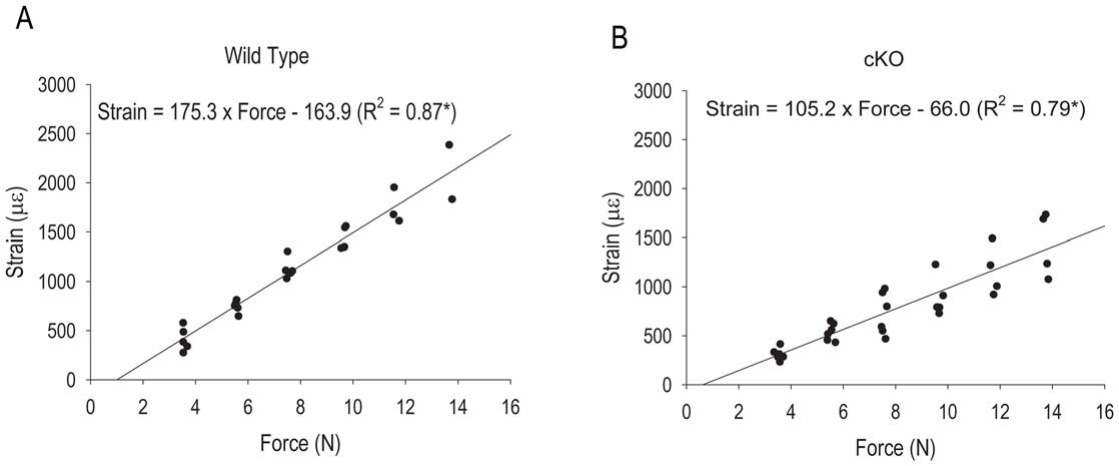
Relationship between applied force and strain measured at the bone surface in (A) wild type and (B) *Gja1* conditional knockout (cKO) mice. Regression equations are given for each genotype, and the slopes of the Regression lines were compared using the test for Parallelism; p<0.05.

Since cKO mice have abnormal cortical structure and biomechanical properties, we first experimentally determined the relationships between applied force and strain generated at the bone surface by axial loading for WT and cKO mice. To this end, the force applied was plotted against the strain detected by strain gauges placed on the surface of the tibia. A regression line was calculated by fitting all experimental data points obtained for each animal in each genotype group. The slope of this regression line was significantly lower for cKO than for WT mice (t = 4.328; p<0.01), implying that cKO bones require greater force to generate the same peak tensile periosteal strain relative to WT mice (Figure 1A, B).

Based on the force-strain plots we used a periosteal strain of 1200 µε for the following studies. This level of strain represents a good compromise between the need of generating an anabolic stimulus and delivering submaximal force. To achieve a periosteal strain of 1200 µε required an axial load of 8 N in the WT (WT-1200 µε) and 12 N in the cKO (cKO-1200 µε). As additional control, a second group of WT mice was loaded at the same force as the cKO, i.e. 12 N, resulting in a periosteal strain magnitude of approximately 1900 µε (WT-1900 µε). Unilateral tibial loading was applied daily (60 cycles for 5 days). Perhaps because of reduced bone strength in cKO mice application of 12 N force in axial compression resulted in tibial fractures in 7 out of 15 cKO mice. Notably, this occurred in mice weighing less than 15 g and these mice were excluded from the analyses. Indeed, *Gja1* cKO mice weighed significantly less than WT mice (16.69±2.77 g vs. 24.54±2.30 g, respectively; p<0.001). The WT-1900 µε mice lost body weight over the 5 day period (−4.53±1.89%; p = 0.047), while the weight loss in the cKO-1200 µε group barely missed the significance level (−7.19±2.55%; p = 0.054). Such degree of weight loss is consistent with a previous study using the same loading regime [14]. There was no significant weight change in the group of WT mice loaded at 1200 µε (0.14±0.81%; p>0.05).

Assessment of trabecular parameters at baseline by in vivo µCT revealed no significant differences between right and left tibiae within each genotype, or between genotypes (not shown). On the other hand, analysis of cortical bone at baseline confirmed the larger marrow (Ma.Ar) and total tissue (Tt.Ar) areas, and decreased cortical bone volume (Ct.BV) and cortical tissue area fraction (Ct.Ar/Tt.Ar) in cKO relative to WT littermates, while there were no differences between controlateral tibiae in each genotype group (Figure S2). Also as previously noted [10], the calculated area moment of inertia was significantly greater in the cKO compared to WT mice (0.106±0.004 mm^4^ vs. 0.069±0.006 mm^4^, respectively; p<0.001).

**Figure 2:**
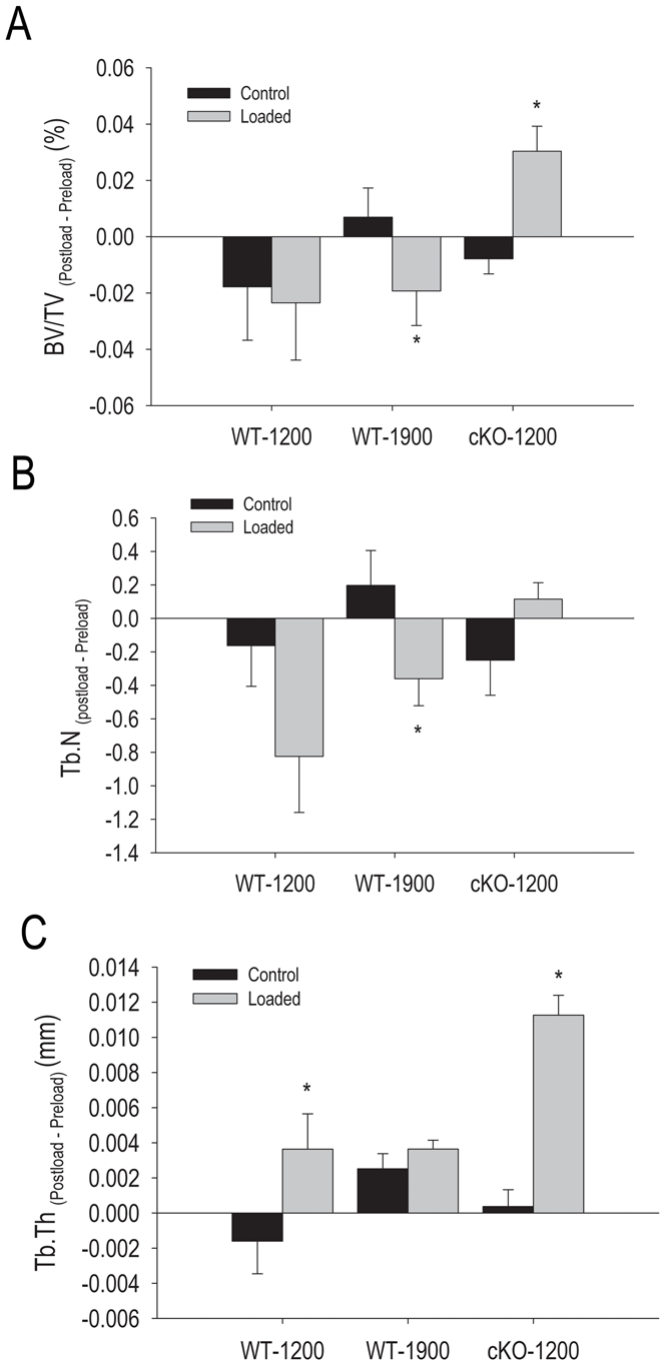
Effect of axial tibial loading on trabecular bone parameters in wild type (WT) and *Gja1* conditional knockout (cKO) mice. The right tibia was subjected to forces generating 1200 µε or 1900 µε in WT, or 1200 µε in cKO, as noted, while the left was used as non-loaded control. (A) Trabecular bone volume (BV/TV); (B) Trabecular Number (Tb.N); (C) Trabecular thickness (Tb.Th). Data are expressed as absolute difference between post- and pre-load for each parameter. *p<0.05 vs. respective non-loaded control; two-tailed t-test.

Application of axial loads that produced either 1200 or 1900 µε strain in WT mice had no stimulatory effect on trabecular bone volume; indeed, we detected a paradoxical decrease in BV/TV at the higher strain. By contrast, 1200 µε was sufficient to generate a significant increase in BV/TV in cKO mice (Figure 2A). No effects of axial load on trabecular number was recorded at 1200 µε in either WT or cKO mice, but there was a decrease at 1900 µε (Figure 2B) consistent with the decrease in BV/TV. In contrast we observed an increase in trabecular thickness in WT mice at 1200 µε, but no effects at the higher strain. Since the latter is the only significant difference in the WT-1200 group, it most likely represents a random finding rather than an effect of load. On the other hand, trabecular thickness increased significantly in the cKO group (Figure 2C).

**Figure 3:**
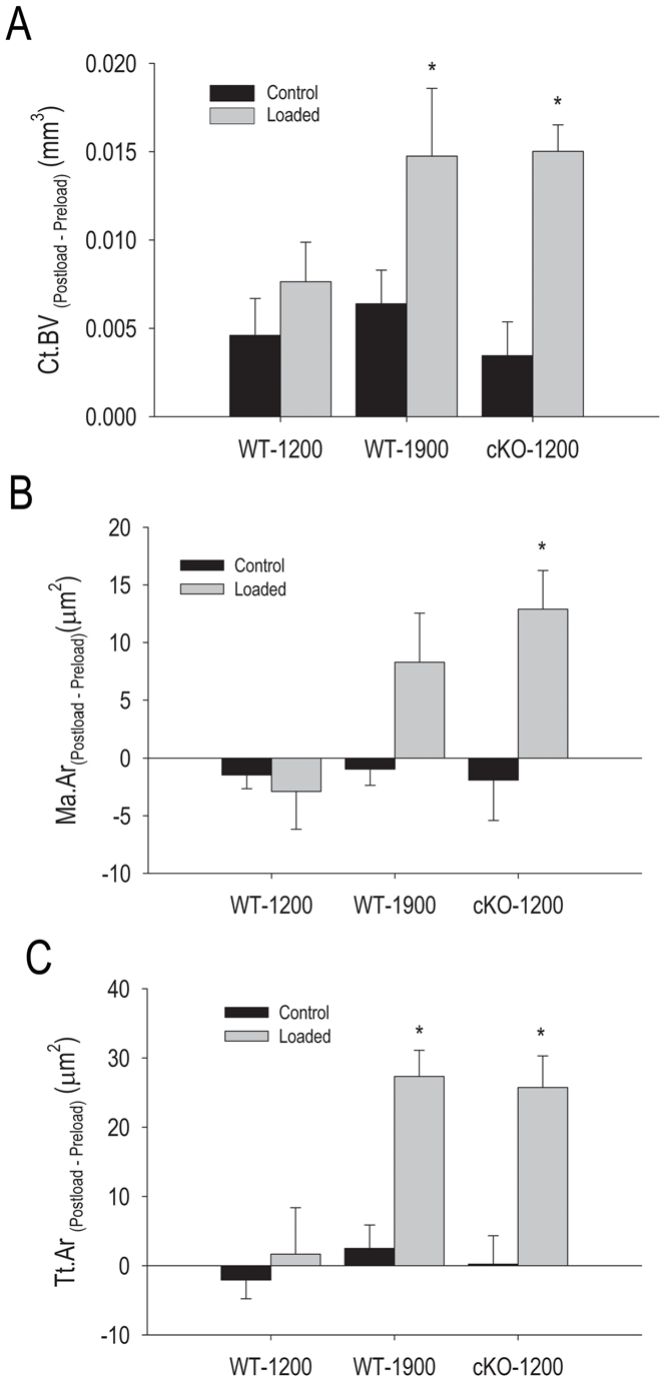
Effect of axial tibial loading on cortical parameters in wild type (WT) and *Gja1* conditional knockout (cKO) mice. The right tibia was subjected to forces generating 1200 µε or 1900 µε in WT, or 1200 µε in cKO, as noted, while the left was used as non-loaded control. (A) Cortical bone volume (Ct.BV). (B) Marrow area (Ma.Ar). (C) Total tissue area (Tt.Ar). Data are expressed as absolute difference between post- and pre-load for each parameter. *p<0.05 vs. respective control; two-tailed t-test.

There was a significant stimulatory effect of loading on cortical bone volume in the WT-1900 and cKO-1200 groups, but not in the WT-1200 group, relative to their respective non-loaded control limbs (Figure 3A). Changes in marrow area and total tissue area after loading followed a similar pattern, with significant increases only in the WT-1900 and cKO-1200 groups and not in the WT-1200 group (Figure 3B, C). These relative changes indicated accrual of periosteal bone with resorption of endocortical bone in the WT-1900 and cKO-1200 groups.

**Figure 4:**
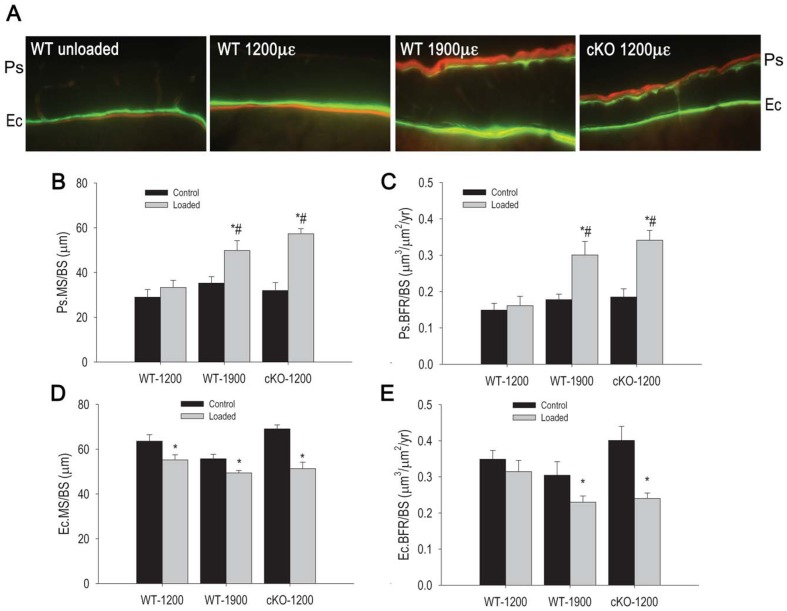
Effect of axial tibial loading on periosteal and endocortical bone formation in wild type (WT) and *Gja1* conditional knockout (cKO) mice. The right tibia was subjected to forces generating 1200 µε or 1900 µε in WT, or 1200 µε in cKO, as noted, while the left was used as non-loaded control. (A) Double calcein/alizarin red labels on cortical sections of tibiae from the different groups, showing both periosteal (Ps) and endocortical (Ec) surfaces. (B) Periosteal mineralizing surfaces per bone surface (Ps.MS/BS) and (C) periosteal bone formation rate per bone surface (Ps.BFR/BS). (D) Endocortical MS/BS (Ec.MS/BS), and (E) endocortical BFR/BS (Ec.BFR/BS). * p<0.05 vs respective Control; # p<0.05 vs WT-1200 Loaded; ANOVA.

Consistent with increased cortical bone volume and cortical total bone area, we detected significant effects of load on periosteal mineralizing surfaces and bone formation rate in the WT-1900 and cKO-1200 groups, but not in the WT-1200 group (Figure 4A–C). On the other hand, at the endocortical surface both indices of bone formation paradoxically decreased after load in both WT groups and such decrease was of significantly larger magnitude in cKO relative to the WT-1200 group (Figures 4D–E and S3). We observed only upward trends in periosteal mineral apposition rate in WT-1900 and cKO-1200, but the differences with control tibiae failed to reach significance; and endocortical mineral apposition rate decreased only in cKO- 1200 but not in WT mice (Figure S4A, B). There were no significant differences in bone formation indices at either periosteal or endocortical surfaces in control tibiae among all groups (Figures 4B–D, and S4).

**Figure 5:**
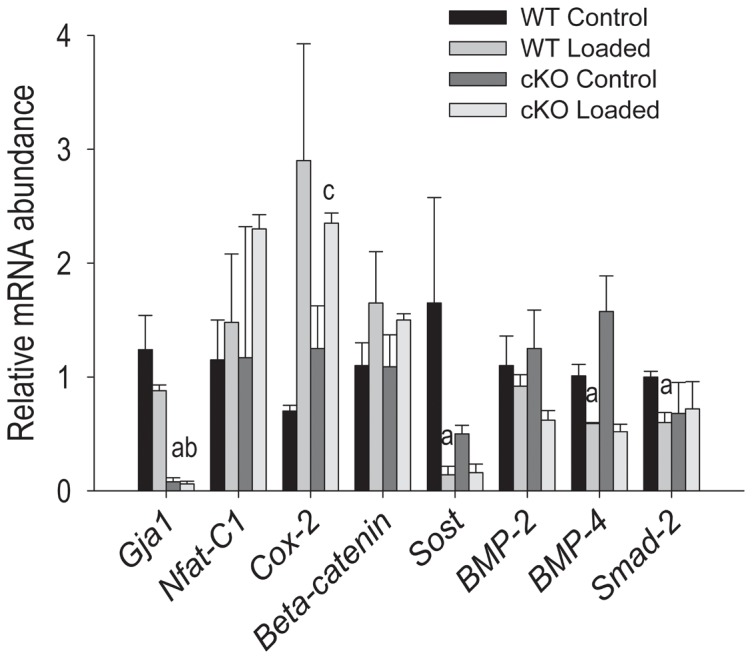
Effect of axial tibial loading on gene expression in vivo. Mice were sacrificed 2 hours after load (120 cycles, 10 sec interval between cycles), and mRNA extracted from whole bone extracts before qPCR analyses for the genes of interest. a: p<0.05 vs WT control; b: p<0.05 vs WT Loaded and c: p<0.05 vs cKO Control; one-way ANOVA.

We next explored expression of genes that had been previously shown to be altered by mechanical load in vitro or in vivo [21]–[23]. Considering the high frequency of fractures in the cKO at 12 N of axial load, we chose to use a lower force, on the assumption that changes in gene expression would be easier to detect at lower loads than changes in osteoblast activity. Thus, we applied a force of 7 N, which generated an estimated periosteal strain of 1063 µε in WT and 670 µε in the cKO. As shown in Fig. 5, we verified that *Gja1* mRNA was barely detectable in the cKO bones using qPCR in marrow-free whole bone extracts. In WT mice the abundance of *Cox-2* mRNA increased 3-fold compared to a doubling in cKO mice, whereas *β-catenin* and *Nfat-C1* mRNA were only marginally increased after mechanical stimulation in both genotypes (Figure 5). Importantly, *Sost* mRNA abundance was dramatically down-regulated by mechanical loading in the WT mice. In cKO mice, *Sost* mRNA was already 50% lower relative to WT at baseline and it was further down-regulated by mechanical load. *Bmp4* mRNA was also significantly down-regulated by mechanical loading in both genotypes, while only negative, non-significant trends were observed for *Bmp2* and *Smad2* mRNA.

## Discussion

This study demonstrates that mice with *Gja1* ablation in the osteogenic lineage activate periosteal bone formation at a lower level of strain than do WT mice, suggesting an increased sensitivity to mechanical load in Cx43 deficiency. Consistently, trabecular bone mass also increases in mutant mice upon load, while it decreases in WT. On the other hand, bone formation actually decreases on the endocortical surface in WT mice upon application of axial mechanical load, and this response is also accentuated in cKO mice. Thus, the response of bone forming cells to mechanical load differs between trabecular and cortical components, and remarkably between endocortical and periosteal envelopes. Cx43 deficiency enhances both the periosteal and endocortical response to mechanical load applied as axial compression in growing mice.

The greater load required in conditional Cx43 deficient mice to generate a specific strain relative to WT mice is consistent with a previous finding using a 3-point bending method to apply load [9]; a finding later confirmed by other groups using different promoters to delete *Gja1* [12], [24]. The abnormal force-strain relationship is most likely related to both the different geometry and altered bone matrix and mineralization of Cx43 deficient bones. The typical phenotype of expanded marrow cavity and periosteal perimeter, particularly evident in the cKO model used in this study, result in a greater area moment of inertia [9], [11], hence a greater resistance to bending in the anterior/posterior direction and lower values of strain for a given compressive load. Strain values upon loading also depend on the bone modulus, which is a function of both the mineral and collagen phases of bone, and both mineral content and collagen fiber organization are abnormal in cKO compared to WT mice [10]. Finally, cKO bones are slightly shorter than WT bones [10], and this could affect response to load. Nonetheless, because we physically measured strains using strain gauges, both geometric and material property differences should be accounted for.

Our data demonstrate that different responses to mechanical loading occur on the periosteal and endocortical bone surfaces. In particular, we find that Cx43 deficiency enhances the periosteal bone formation response to loading (for equivalent strains) and further decreases bone formation on the endocortical response relative to WT bones in growing mice. This differential response explains the apparently discordant results obtained by independent studies that have examined the different cortical envelopes [9], [12]. The characteristics of the loading regime may result in different strain stimuli at different sites and thus in different outcomes. Axial tibial compression loading produces both a compressive and bending load on the tibia due to the curvature of the bone. Although a bit surprising, a negative effect of axial mechanical load on trabecular bone of WT mice is consistent with previous studies from our group in aged mice, showing that trabecular bone volume actually decreases in young adult (7 months) mice upon axial compression using the waveform used here [14]. This is in contrast to findings from other groups who have used tibial compression under different loading regimes [25]–[27]. Preliminary results from our laboratory suggest this discrepancy may depend on the waveform and cycle number. On the other hand, BV/TV significantly increased in cKO mice with 1200 µε load, further suggesting that Cx43 is involved in response to load, though the trabecular compartment in the cKO may experience greater compressive strains than WT due to the altered architecture.

Interestingly, axial load increased periosteal formation rate in cKO loaded at 1200 µε as well as in WT loaded at 1900 µε; however, periosteal mineral apposition rate did not significantly change in either group. Such a discrepancy implies a higher number of osteoblasts depositing new bone, rather than increased activity of existing osteoblasts. Thus, these results are consistent with the notion that axial compression loading promotes osteoblast recruitment and differentiation on the periosteal surface, an effect enhanced by Cx43 deficiency. It is possible that signaling from osteocytes to the periosteal surface is enhanced in the cKO. However, since periosteal cells in cKO mice are exposed to a lower degree of strain for a given force (based on our force-strain analysis), the fact that periosteal bone formation is increased in Cx43 deficient bones relative to WT bone even in animals living under normal loading conditions [24] strongly suggests that periosteal cells from cKO are more sensitive to mechanical strain than are periosteal WT cells. This conclusion is fully consistent with one of the main findings of this study demonstrating that 1200 µε was sufficient in cKO to elicit the same bone formation response as did 1900 µε in WT animals. Notably, a periosteal increase in bone diameter contributes more to load bearing strength than do increases in cortical thickness associated with endocortical apposition [28]. Thus, the increased periosteal bone formation observed in the cKO may be related to the need for generating bone at the diaphysis to withstand loads that are sensed as abnormally higher than they actually are.

Increased periosteal bone apposition but decreased endosteal formation after mechanical loading had been previously reported in 9 week old C3H/HeJ mice [29]. Such a paradoxical effect on the endocortical surface may reflect the young age of the mice used in these studies, and the decrease in bone formation rate may represent a slowing of the rapid bone formation rate occurring in growing mice. Since such an effect is more pronounced in mice with an osteoblast/osteocyte specific deletion of *Gja1*, it can be concluded that Cx43 deficiency also increases (in this case in a negative direction) the sensitivity of the endocortical cells to mechanical loading.

Decreased endocortical bone formation by axial load is in apparent contrast with our previous study showing increased endosteal bone formation after application of a 3-point bending load [9]. Aside the different loading approaches, other differences in the experimental settings may contribute to the discrepancy, including a substantially larger strain (1600 µε at the anterior-lateral endocortical surface, corresponding to 3774 µε at the periosteal surface) and older mice (4-month-old) used in the 3-point bending experiment relative to the present study, as well as different promoters used to drive *Gja1* ablation. In both cases, however, Cx43 deficiency reduces bone formation at the endocortex upon mechanical loading. Lack of differences in periosteal bone formation between WT and cKO at baseline is also in apparent contrast with our previous report [10]. However in that study surface labeling was assessed from the proximal to distal ends of the tibia, whereas in the present experiments cross sections were cut close to the mid-diaphysis – the location at which there is least bone formation activity, and thus genotype differences may be minimized. Consistent with a previous study from our groups using the same loading regime [14], there was a modest weight loss at the end of the study, but this was only significant in one group, WT-1900, which also lost cancellous bone mass. However, cortical bone mass and bone formation rates increased in these mice, and trabecular bone volume increased in the cKO-1200 group, which experienced an even higher weight loss. Therefore it is unlikely that weight loss may have substantially affected changes in bone mass.

While the mechanisms underlying the enhanced periosteal sensitivity require more in-depth investigation, our gene expression studies point to a potentially significant role of sclerostin, the product of *Sost*, a paracrine inhibitor of Wnt signaling. The robust down-regulation of *Sost* expression, among all the genes we investigated, in both WT and cKO implies activation of Wnt signaling by mechanical load. Indeed, the anabolic response to mechanical load is dependent on an intact Lrp5/β-catenin signaling system [21], [30], and β-catenin abundance or activity is affected by mechanical stimulation [31], [32]. Furthermore, *Sost* is up-regulated by skeletal unloading [33]. Hence, *Sost* down-regulation in Cx43 deficient animals, a finding we had already reported [10], and recently confirmed by others [24], perfectly fits with the model of increased sensitivity of Cx43 deficient bones to mechanical load. On the other hand, this model does not explain the decreased endocortical bone formation upon loading and the further decrease in cKO mice. A very recent study proposes that sclerostin production is altered by Cx43 deficiency specifically in osteocytes in close proximity to the periosteal surface [24]. However, unless one postulates a selective flow of sclerostin from matrix embedded osteocytes to the periosteal surface, an autonomous defect in mechanosensing by periosteal and endocortical cells remains a viable hypothesis.

We also observed the expected increase in *Cox2* expression after axial tibial compression, and this effect was attenuated in Cx43 cKO mice. Although this might reflect a low degree of strain applied to the cKO bones, it is very likely that the cycloxygenase pathway is integrated in the mechanotransduction mechanism modulated by Cx43 [34]. Recent work from our group suggests that the Wnt and BMP signaling systems reciprocally control cell proliferation in bone [35]; thus, the decrease of *Bmp4*, and to a lesser extent *Bmp2*, together with *Sost* down-regulation we observed in this study might be seen as part of a general response to mechanical loading, resulting in expansion of bone forming cells and increased periosteal bone formation.

In summary, we show that Cx43 deficiency in bone cells alters the relationship between mechanical force applied as axial compression and strain produced on the bone surface, so that a higher force is required to generate the same amount of strain compared to WT bones. Importantly, we demonstrate that a lower degree of strain is required to stimulate periosteal bone formation in Cx43 deficient mice, and that this is associated with profound down-regulation of *Sost* and increased *Cox2* mRNA expression. Furthermore, we find that contrary to the stimulatory effect on the periosteal surface, axial tibial mechanical load does not activate endocortical bone formation, and it actually decreases it in conditions of Cx43 deficiency. Thus, Cx43 regulates cortical bone remodeling in part by modulating bone cell sensitivity to mechanical load.

## Supporting Information

Figure S1 X-gal stained, paraffin-embedded section of the diaphysis of *Gja1* conditional knockout mouse, counterstained with H&E. Strong β-galactosidase activity is evident in periosteal cells and, to a lesser degree, in osteocytes.
(TIF)

Figure S2 Baseline cortical bone morphology of wild type (WT) and *Gja1* conditional knockout mice (cKO). The right tibia was subjected to forces generating 1200 µε or 1900 µε in WT, or 1200 µε in cKO, as noted, while the left was used as non-loaded control. (A) Marrow area (Ma.Ar). (B) Total tissue area (Tt.Ar). (C) Cortical bone volume (Ct.BV). (D) Cortical area/total tissue area (Ct.Ar/Tt.Ar). * p<0.05 vs WT-1200 and WT-1900 (one-way ANOVA).
(TIF)

Figure S3 Percent changes in endocortical bone formation rate per bone surface (Ec.BFR/BS) in loaded relative to control bones after application of axial load. The decrease in Ec.BFR/BS was significantly larger in the *Gja1* conditional knockout (cKO) than in the WT-1200 group (p<0.05; two-tailed t-test); whereas the difference between WT-1900 and cKO-1200 was not statistically significant.
(TIF)

Figure S4 Effect of axial tibial loading on periosteal (A) and endocortical (B) mineral apposition rate (MAR) in wild type (WT) and *Gja1* conditional knockout (cKO) mice. The right tibia was subjected to forces generating 1200 µε or 1900 µε in WT, or 1200 µε in cKO, as noted, while the left was used as a non-loaded control. *p<0.05 vs. respective control; two-tailed t-test.
(TIF)

Table S1 Forward and reverse primers used for real time PCR in the gene expression analyses for wild type (WT) and *Gja1* conditional knockout (cKO) mice loaded at 7 N in axial tibial compression loading.
(DOCX)

## References

[1] Civitelli R, Beyer EC, Warlow PM, Robertson AJ, Geist ST, et al (1993) Connexin43 mediates direct intercellular communication in human osteoblastic cell networks. J Clin Invest 91: 1888–1896. 8387535 10.1172/JCI116406 PMC288182

[2] Van der Molen MA, Rubin CT, McLeod KJ, McCauley LK, Donahue HJ (1996) Gap junctional intercellular communication contributes to hormonal responsiveness in osteoblastic networks. J Biol Chem 271: 12165–12171. 8647809 10.1074/jbc.271.21.12165

[3] Civitelli R (2008) Cell-cell communication in the osteoblast/osteocyte lineage. Arch Biochem Biophys 473: 188–192. 18424255 10.1016/j.abb.2008.04.005 PMC2441851

[4] Lecanda F, Warlow PM, Sheikh S, Furlan F, Steinberg TH, et al (2000) Connexin43 deficiency causes delayed ossification, craniofacial abnormalities, and osteoblast dysfunction. J Cell Biol 151: 931–944. 11076975 10.1083/jcb.151.4.931 PMC2169447

[5] Paznekas WA, Boyadjiev SA, Shapiro RE, Daniels O, Wollnik B, et al (2003) Connexin 43 (GJA1) mutations cause the pleiotropic phenotype of oculodentodigital dysplasia. AmJHumGenet 72: 408–418. 10.1086/346090 PMC379233 12457340

[6] Kjaer KW, Hansen L, Eiberg H, Leicht P, Opitz JM, et al (2004) Novel Connexin 43 (GJA1) mutation causes oculo-dento-digital dysplasia with curly hair. AmJMedGenet 127A: 152–157. 10.1002/ajmg.a.20614 15108203

[7] Malone AM, Anderson CT, Tummala P, Kwon RY, Johnston TR, et al (2007) Primary cilia mediate mechanosensing in bone cells by a calcium-independent mechanism. Proc Natl Acad Sci U S A 104: 13325–13330. 17673554 10.1073/pnas.0700636104 PMC1939687

[8] Chung DJ, Castro CH, Watkins M, Stains JP, Chung MY, et al (2006) Low peak bone mass and attenuated anabolic response to parathyroid hormone in mice with an osteoblast-specific deletion of connexin43. J Cell Sci 119: 4187–4198. 16984976 10.1242/jcs.03162

[9] Grimston SK, Brodt MD, Silva MJ, Civitelli R (2008) Attenuated response to in vivo mechanical loading in mice with conditional osteoblast ablation of the connexin43 gene (Gja1). J Bone Miner Res 23: 879–886. 18282131 10.1359/JBMR.080222 PMC2677086

[10] Mahajan G, Kotru M, Batra M, Gupta A, Sharma S (2011) Usefulness of histopathological examination in uterine prolapse specimens. The Australian & New Zealand journal of obstetrics & gynaecology 51: 403–405. 21810084 10.1111/j.1479-828X.2011.01337.x

[11] Grimston SK, Goldberg DB, Watkins M, Brodt MD, Silva MJ, et al (2011) Connexin43 deficiency reduces the sensitivity of cortical bone to the effects of muscle paralysis. J Bone Miner Res 10.1002/jbmr.425 PMC3306012 21590735

[12] Zhang Y, Paul EM, Sathyendra V, Davison A, Sharkey N, et al (2011) Enhanced osteoclastic resorption and responsiveness to mechanical load in gap junction deficient bone. PLoS One 6: e23516. 21897843 10.1371/journal.pone.0023516 PMC3163577

[13] Truett GE, Heeger P, Mynatt RL, Truett AA, Walker JA, et al (2000) Preparation of PCR-quality mouse genomic DNA with hot sodium hydroxide and tris (HotSHOT). Biotechniques 29: 52, 54. 10907076 10.2144/00291bm09

[14] Brodt MD, Silva MJ (2010) Aged mice have enhanced endocortical response and normal periosteal response compared with young-adult mice following 1 week of axial tibial compression. J Bone Miner Res 25: 2006–2015. 20499381 10.1002/jbmr.96 PMC3153404

[15] Christiansen BA, Bayly PV, Silva MJ (2008) Constrained tibial vibration in mice: a method for studying the effects of vibrational loading of bone. J Biomech Eng 130: 044502. 18601464 10.1115/1.2917435 PMC2893880

[16] Silva MJ, Brodt MD, Hucker WJ (2005) Finite element analysis of the mouse tibia: estimating endocortical strain during three-point bending in SAMP6 osteoporotic mice. AnatRecA DiscovMolCell EvolBiol 283: 380–390. 10.1002/ar.a.20171 15747345

[17] Parfitt AM, Drezner MK, Glorieux FH, Kanis JA, Malluche H, et al (1987) Bone histomorphometry: standardization of nomenclature, symbols, and units. Report of the ASBMR Histomorphometry Nomenclature Committee. J Bone Miner Res 2: 595–610. 3455637 10.1002/jbmr.5650020617

[18] Foldes J, Shih MS, Parfitt AM (1990) Frequency distributions of tetracycline-based measurements: implications for the interpretation of bone formation indices in the absence of double-labeled surfaces. J Bone Miner Res 5: 1063–1067. 2080717 10.1002/jbmr.5650051010

[19] Stains JP, Civitelli R (2003) Genomic approaches to identifying transcriptional regulators of osteoblast differentiation. Genome Biol 4: 222. 12844353 10.1186/gb-2003-4-7-222 PMC193624

[20] Mbalaviele G, Sheikh S, Stains JP, Salazar VS, Cheng SL, et al (2005) β-catenin and BMP-2 synergize to promote osteoblast differentiation and new bone formation. J Cell Biochem 94: 403–418. 15526274 10.1002/jcb.20253 PMC2647989

[21] Robinson JA, Chatterjee-Kishore M, Yaworsky PJ, Cullen DM, Zhao W, et al (2006) Wnt/beta-catenin signaling is a normal physiological response to mechanical loading in bone. J Biol Chem 281: 31720–31728. 16908522 10.1074/jbc.M602308200

[22] Forwood MR (1996) Inducible cyclo-oxygenase (COX-2) mediates the induction of bone formation by mechanical loading in vivo. J Bone Miner Res 11: 1688–1693. 8915776 10.1002/jbmr.5650111112

[23] Gluhak-Heinrich J, Gu S, Pavlin D, Jiang JX (2006) Mechanical loading stimulates expression of connexin 43 in alveolar bone cells in the tooth movement model. Cell CommunAdhes 13: 115–125. 10.1080/15419060600634619 PMC1797153 16613785

[24] Bivi N, Condon KW, Allen MR, Farlow N, Passeri G, et al (2011) Cell autonomous requirement of connexin 43 for osteocyte survival: consequences for endocortical resorption and periosteal bone formation. J Bone Miner Res 27: 374–389. 10.1002/jbmr.548 PMC3271138 22028311

[25] Fritton JC, Myers ER, Wright TM, van der Meulen MC (2005) Loading induces site-specific increases in mineral content assessed by microcomputed tomography of the mouse tibia. Bone 36: 1030–1038. 15878316 10.1016/j.bone.2005.02.013

[26] Fritton JC, Myers ER, Wright TM, van der Meulen MC (2008) Bone mass is preserved and cancellous architecture altered due to cyclic loading of the mouse tibia after orchidectomy. J Bone Miner Res 23: 663–671. 18433300 10.1359/JBMR.080104 PMC2674541

[27] Sugiyama T, Saxon LK, Zaman G, Moustafa A, Sunters A, et al (2008) Mechanical loading enhances the anabolic effects of intermittent parathyroid hormone (1–34) on trabecular and cortical bone in mice. Bone 43: 238–248. 18539556 10.1016/j.bone.2008.04.012

[28] De Souza RL, Matsuura M, Eckstein F, Rawlinson SC, Lanyon LE, et al (2005) Non-invasive axial loading of mouse tibiae increases cortical bone formation and modifies trabecular organization: a new model to study cortical and cancellous compartments in a single loaded element. Bone 37: 810–818. 16198164 10.1016/j.bone.2005.07.022

[29] Wyatt SS, Price RA, Holthouse D, Elsaleh H (2001) Choroid plexus carcinoma in an adult. Australasian radiology 45: 369–371. 11531769 10.1046/j.1440-1673.2001.00941.x

[30] Sawakami K, Robling AG, Ai M, Pitner ND, Liu D, et al (2006) The Wnt co-receptor LRP5 is essential for skeletal mechanotransduction but not for the anabolic bone response to parathyroid hormone treatment. J Biol Chem 281: 23698–23711. 16790443 10.1074/jbc.M601000200

[31] Norvell SM, Alvarez M, Bidwell JP, Pavalko FM (2004) Fluid shear stress induces beta-catenin signaling in osteoblasts. Calcif Tissue Int 75: 396–404. 15592796 10.1007/s00223-004-0213-y

[32] Hens JR, Wilson KM, Dann P, Chen X, Horowitz MC, et al (2005) TOPGAL mice show that the canonical Wnt signaling pathway is active during bone development and growth and is activated by mechanical loading in vitro. J Bone Miner Res 20: 1103–1113. 15940363 10.1359/JBMR.050210

[33] Lin C, Jiang X, Dai Z, Guo X, Weng T, et al (2009) Sclerostin mediates bone response to mechanical unloading through antagonizing Wnt/beta-catenin signaling. J Bone Miner Res 24: 1651–1661. 19419300 10.1359/jbmr.090411

[34] Bonewald LF, Johnson ML (2008) Osteocytes, mechanosensing and Wnt signaling. Bone 42: 606–615. 18280232 10.1016/j.bone.2007.12.224 PMC2349095

[35] Salazar V, Zarkadis N, Huang L, Mbalaviele G, Civitelli R (2011) Smad4 Antagonizes Osteoblast Proliferation via Competitive Recruitment of beta-catenin. 33rd Annual Meeting of the American Society for Bone and Mineral Research, San Diego, California, USA.

