# Supplementary material for: Enhanced Periosteal and Endocortical Responses to Axial Tibial Compression Loading in Conditional Connexin43 Deficient Mice
Source: PLoS One. 2012 Sep 10;7(9):e44222. doi: 10.1371/journal.pone.0044222 (PMC3438198; doi:10.1371/journal.pone.0044222)
Supplement: Table S1 — Forward and reverse primers used for real time PCR in the gene expression analyses for wild type (WT) and Gja1 conditional knockout (cKO) mice loaded at 7 N in axial tibial compression loading. (DOCX) [file pone.0044222.s005.docx]

Supplemental Table 1: List of forward and reverse primers used for qPCR analyses

|  | Forward Primer | Reverse Primer |
| --- | --- | --- |
| *BMP-2* | GGGACCCGCTGTCTTCTAGT | TCAACTCAAATTCGCTGAGGAC |
| *BMP-4* | TTCCTGGTAACCGAATGCTGA | CCTGAATCTCGGCGACTTTTT |
| β*-catenin* | GCCGAGATGGCCCAGAA | GATGGTGGGTGCAGGAGTTTAA |
| *Cox-2* | GGGTTGCTGGGGGAAGAAATGTG | CTCTCTGCTCTGGTCAATGG |
| *Cphn (Ppia)* | AGCATACAGGTCCTGGCATC | TTCACCTTCCCAAAGACCAC |
| *Gja1* | CGGTTGTGAAAATGTCTGCTATG | GGCACAGACACGAATATGATCTG |
| *Nfat C1* | CCCGTCACATTCTGGTCCAT | CAAGTAACCGTGTAGCTGCACAA |
| *Smad2* | ATGTCGTCCATCTTGCCATTC | AACCGTCCTGTTTTCTTTAGCTT |
| *Sost* | AGCCTTCAGGAATGATGCCAC | CTTTGGCGTCATAGGGATGGT |
